# Digital Health Transition in Rheumatology: A Qualitative Study

**DOI:** 10.3390/ijerph18052636

**Published:** 2021-03-05

**Authors:** Felix Mühlensiepen, Sandra Kurkowski, Martin Krusche, Johanna Mucke, Robert Prill, Martin Heinze, Martin Welcker, Hendrik Schulze-Koops, Nicolas Vuillerme, Georg Schett, Johannes Knitza

**Affiliations:** 1 Center for Health Services Research, Brandenburg Medical School Theodor Fontane, 15562 Rüdersdorf, Germany; martin.heinze@immanuelalbertinen.de; 2 Faculty of Health Sciences Brandenburg, Brandenburg Medical School Theodor Fontane, 14476 Potsdam, Germany; Robert.Prill@mhb-fontane.de; 3 Department of Palliative Medicine, CCC Erlangen-EMN, Universitätsklinikum Erlangen, Friedrich-Alexander-Universität Erlangen-Nürnberg (FAU), 91054 Erlangen, Germany; Sandra.Kurkowski@uk-erlangen.de; 4 Rheumatology and Clinical Immunology, Charité Universitätsmedizin Berlin, 10117 Berlin, Germany; martin.krusche@charite.de; 5 Policlinic and Hiller Research Unit for Rheumatology, Heinrich-Heine-University, 40225 Duesseldorf, Germany; Johanna.Mucke@med.uni-duesseldorf.de; 6 Department of Orthopaedics and Trauma Surgery, Brandenburg Medical School Theodor Fontane, Municipal Clinic Brandenburg, 14770 Brandenburg, Germany; 7 Department of Psychiatry and Psychotherapy, Brandenburg Medical School Theodor Fontane, Immanuel Klinik Rüdersdorf, 15562 Rüdersdorf, Germany; 8 Medizinisches Versorgungszentrum für Rheumatologie Dr. M. Welcker GmbH, 82152 Planegg, Germany; Martin.Welcker@rheumatologie-welcker.de; 9 Division of Rheumatology and Clinical Immunology, Department of Medicine IV, Ludwig-Maximilians-Universität München, 80336 Munich, Germany; Hendrik.Schulze-Koops@med.uni-muenchen.de; 10 AGEIS, Faculty of Medicine, Université Grenoble Alpes, 38706 Grenoble, France; Nicolas.Vuillerme@univ-grenoble-alpes.fr (N.V.); johannes.knitza@uk-erlangen.de (J.K.); 11 Institut Universitaire de France, 75006 Paris, France; 12 LabCom Telecom4Health, Université Grenoble Alpes & Orange Labs, 38400 Grenoble, France; 13 Department of Internal Medicine 3—Rheumatology and Immunology, Friedrich-Alexander University Erlangen-Nürnberg and Universitätsklinikum Erlangen, 91054 Erlangen, Germany; georg.schett@uk-erlangen.de; 14 Deutsches Zentrum für Immuntherapie, Friedrich-Alexander University Erlangen-Nürnberg and Universitätsklinikum Erlangen, 91054 Erlangen, Germany

**Keywords:** rheumatology, chronic disease, digital health, eHealth, telemedicine, remote care, patient perspective, qualitative research, fishbowl discussion, content analysis

## Abstract

The global COVID-19 pandemic has led to drastic changes in the management of patients with rheumatic diseases. Due to the imminent risk of infection, monitoring intervals of rheumatic patients have prolonged. The aim of this study is to present insights from patients, rheumatologists, and digital product developers on the ongoing digital health transition in rheumatology. A qualitative and participatory semi-structured fishbowl approach was conducted to gain detailed insights from a total of 476 participants. The main findings show that digital health and remote care are generally welcomed by the participants. Five key themes emerged from the qualitative content analysis: (1) digital rheumatology use cases, (2) user descriptions, (3) adaptation to different environments of rheumatology care, and (4) potentials of and (5) barriers to digital rheumatology implementation. Codes were scaled by positive and negative ratings as well as on micro, meso, and macro levels. A main recommendation resulting from the insights is that both patients and rheumatologists need more information and education to successfully implement digital health tools into clinical routine.

## 1. Introduction

COVID-19 has led to drastic changes in the management of patients with rheumatic diseases. Due to the imminent risk of infection, monitoring intervals of rheumatic patients have prolonged [1,2]. Some were cancelled or postponed, whereas others were replaced by video consultations [1,3], thereby catalyzing digital disruption in healthcare. Besides the COVID-19-induced uptake of digital health [4], the Digital Health Act, passed in December 2019, reshapes the German healthcare system [5]. Measures include the introduction of digital health applications into the statutory health insurance scheme, the implementation of digital patient records, and the promotion of video consultation hours [3,5]. Due to aggravating challenges, such as the declining number of rheumatologists [6], the aging population, and the need for early diagnosis [7] and continuous monitoring [8], rheumatology is considered to have a great potential to benefit from a digital health transition [2,9].

In a response to the current challenges and uptake of digitization, a virtual fishbowl discussion was organized at the annual scientific conference of the German Society of Rheumatology (DGRh Congress), which took place in September 2020. Patients, rheumatologists, and industry stakeholders had the opportunity to exchange information and experiences on digital health in rheumatology. The aim of this study is to present insights from patients, rheumatologists, and digital product developers on the ongoing digital health transition and innovation potentials in rheumatology in Germany.

## 2. Materials and Methods

The authors conducted a virtual fishbowl discussion [10] on the question “How does the internet affect the doctor–patient relationship?” at the first virtual annual conference of the German Society for Rheumatology 2020 (9–12 September 2020). The event was scheduled for 90 min. It consisted of two introductory key notes on digital health services in rheumatology and the actual fishbowl discussion. The discussion was recorded, transcribed verbatim, and later examined using qualitative content analysis [11].

**Figure 1:**
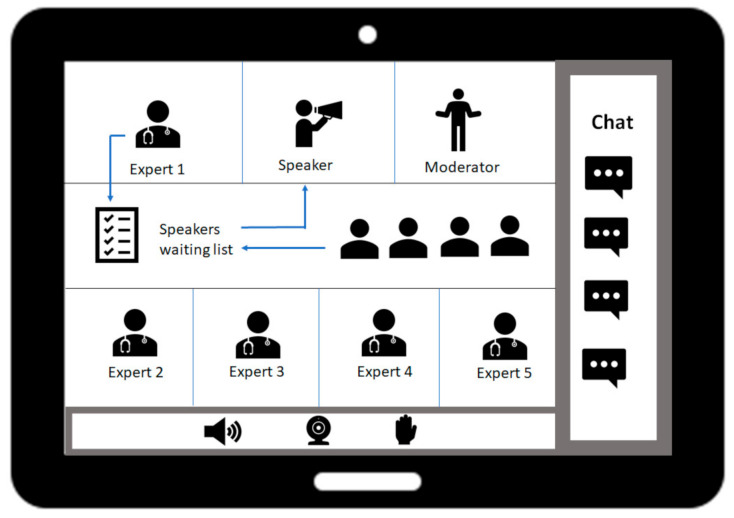
Virtual fishbowl discussion (in accordance with Muehlensiepen et al. [10]).

Recently, the fishbowl technique has been successfully implemented in national rheumatology conferences [12]. It is a validated method, fostering open group discussions and engagement of all audience members [13]. By including an expert panel (inner circle) and one empty chair for an alternating audience member, the technique promotes a dynamic and direct exchange with the audience. The authors intended to hold a face-to-face fishbowl discussion at DGRh Congress 2020. Due to the COVID-19 pandemic, the entire conference was held virtually, using Zoom™-based software (Zoom Video Communications Inc, San Jose, CA, USA) [14]. Hence, the authors successfully tested the fishbowl discussion as an online format (Figure 1).

The inner circle of the fishbowl discussion consisted of one patient representative, two rheumatologists, and two digital product developers. To contribute to the discussion, they used the “hand raise” function to communicate with the moderator (F.M.). Audience members (the outer circle) who wanted to take part in the discussion and take the empty seat in the inner circle had to report this interest in the chat and were registered in the speaker list. When it was their turn, the participants’ cameras and microphones were activated by tech support. After their statement, the participants left the inner circle of the discussion again.

As the fishbowl was part of the official congress program, delegates could also participate in the discussion without taking part in the study: either by not contributing verbally or by indicating that their contribution would be deleted from the recording. However, delegates did not use this option.

**Figure 2:**
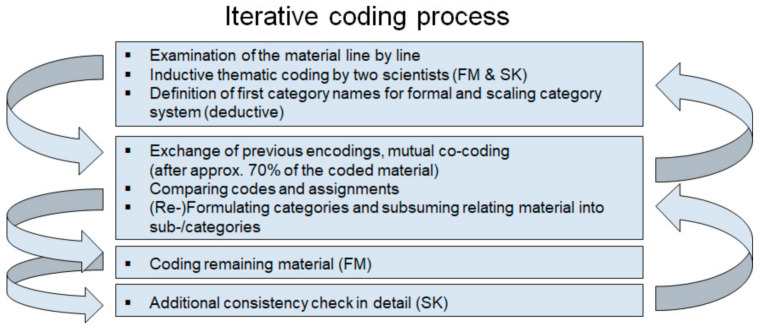
Qualitative content analysis: iterative coding process.

Analysis was based on qualitative content analysis by Philip Mayring [11]. The fishbowl discussion was audio-recorded and subsequently anonymized and transcribed verbatim. The transcript was imported into MAXQDA 2020 Software (VERBI GmbH, Berlin, Germany) [15]. The data analysis involved four steps (Figure 2).

**Figure 3:**
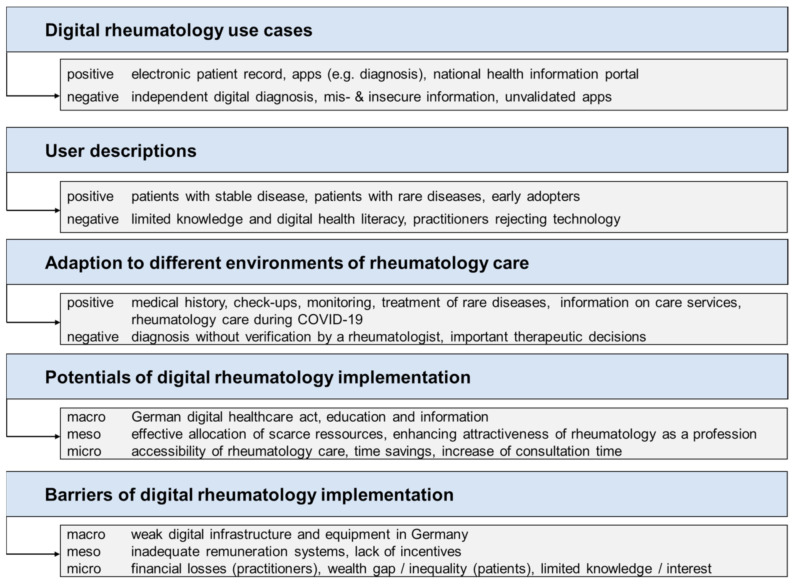
Qualitative content analysis: main categories (according to Kurkowski et al. 2020 [16]).

Step 1: Two scientists with a background in healthcare science (F.M., male, PhD) and social science (S.K., female, M.Sc.) independently examined the material line by line, coded inductively with regard to the content (thematic coding) and formulated as closely to the notes as possible. Additionally, codes were scaled in accordance with Kurkowski et al. [16]. Formal and scaled codes were assigned simultaneously and deductively to matching text sections. Positive and negative connotations were differentiated (scaling). Codes were scaled to the category “positive rating” if they contained clearly positive expressions (e.g., “ideal,” “great opportunity”), whereas the codes were scaled as “negative” if they expressed a clearly negative connotation (e.g., “we do not wish,” “a negative example”). If codes could not be assigned to a positive or negative scaling, they were not considered in the further analysis. Step 2, after approximately 70% of the material was coded, the two scientists (F.M. and S.K.) exchanged their codes and compared their codes and assignments. After this, they defined the main categories and subsumed relating codes. The main categories represent the central themes and aspects of the discussion: (1) digital rheumatology use cases, (2) user descriptions, (3) adaptation to different environments of rheumatology care, and (4) potentials of and (5) barriers to digital rheumatology implementation (Figure 3). The categories, potentials (4), and barriers (5) of digital rheumatology implementation were grouped into micro, meso, and macro levels. Codes were assigned to the macro level if they related to the German health system. The meso level referred to rheumatology care as a whole. The micro level concerned the individual patient–practitioner level. Step 3: After reworking the code system, one scientist (F.M.) coded the remaining notes. Step 4: S.K. performed an additional consistency check, and inconsistencies were resolved. To ensure validity of the data analysis, the results of the analysis were reviewed and confirmed by participants of the fishbowl discussion (M.W., H.S.K., and J.K.) before drafting the manuscript. For the presentation of the results, representative quotes of the discussion transcript were selected, translated, and included in the text. The manuscript has been compiled in accordance with the Consolidated Criteria for Reporting Qualitative Research (COREQ) [17] (please refer to Supplementary Materials 1).

Ethical approval was not sought for the present study as this manuscript reports on the results of a discussion held during a virtual medical conference. Despite this, all authors declared to adhere to the Declaration of Helsinki in its current form in all steps of this project [18]. Discussion participants were informed of the audio recording, transcription, and subsequent analysis of the discussion. In the course of transcription, all data and personal references were removed. There was no risk involved in participating in the discussion. The manuscript does not allow identification of individuals.

## 3. Results

A total of 476 delegates attended the 90-min session. The actual fishbowl discussion lasted 56 min. In total, there were 19 content contributions by 12 delegates: 9 rheumatologists, 2 digital product developers, and 1 patient representative. Seven of the 19 contributions came from audience members from the outer circle (37%).

### 3.1. Digital Rheumatology Use Cases

Delegates discussed various examples of how digital health could complement rheumatology care. Implementation of electronic health records in Germany was considered to have great potential for boosting doctor–patient relationships as well as tracking previous medical care, resulting in more efficient use of scarce time:

*“Electronic patient records—the next big thing. I see this as an extremely great opportunity to strengthen the doctor–patient relationship and this precious therapy time of five minutes and so on.”*

*(Patient representative I)*

Participants pointed out the importance of implementing a digital national health portal where patients could access validated and evidence-based information about their medical conditions:

*“I consider the concept as a chance to provide sensible content to patients, to people, in a centralized way.”*

*(Rheumatologist I)*

The introduction of healthcare apps into German Social Health Insurance system is expected to have great potential and a positive impact on rheumatology care as well:

*“I think this extension and supplementation of care through digital health apps, DiGAs, will be a nice, good, viable addition.”*

*(Digital product developer I)*

*“On the one hand sensitivity and specificity [of diagnosis apps] is very poor at 50 percent. But on the other hand it’s still 20 percent higher than the referral of the non-experienced general practitioner, with a specificity of about 30 percent. That means there’s a clear potential for improvement without any physical examination, as you said, with the methods of [name of a certain digital health application] or [name of another application] or other diagnostic apps. But the power has already been demonstrated.”*

*(Rheumatologist II)*

Delegates also discussed negative examples of digital healthcare in chronic diseases. Approaches were seen as negative when they are disconnected from the “regular” health system and digital healthcare is not controlled by medical practitioners:

*“But that’s what you read now in magazines again and again in a striking way: A patient has foam in her urine, feels somehow bad, weary, and so on, goes to the convenience store, somehow hands in her sample and then receives her results digitally, oh, she knows it’s lupus? That is, I think, not exactly the good start to a chronic patient’s career and good care that we actually want.”*

*(Patient representative I)*

Additionally, delegates identified insecure sources and misinformation on diseases as a danger of digital health. Furthermore, they expressed their opposition to invalidated digital health applications without proof of benefit. Interestingly, video consultations were not a main discussion topic.

### 3.2. User Descriptions

Discussants identified patients with a stable course of their chronic rheumatic disease as potential users of digital health tools:

*“And, for a well-cared for or well-adjusted patient like me, a doctor’s contact is really necessary only once a year for therapy monitoring reasons, and if there is something beyond that, like maybe checking whether my blood pressure needs to be adjusted, talking to the doctor once in a while, that would of course be ideal to cover the perhaps necessary two to three doctor’s contacts a year once briefly via a secure telemedicine platform.”*

*(Patient representative I)*

Digital health can also significantly help considering rare diseases:

*“Particularly in the area of rare diseases or unusual symptoms, patients also do research on the internet if they do their research well, find good sources or do research at organizations or patient self-help organizations. Patients who use swarm intelligence can also arrive more quickly at more or less target-oriented diagnostic suggestions. That is a knowledge gain overall.”*

*(Patient representative I)*

Among physicians, early adopters were mentioned as potential users of digital health. Less technophile rheumatologists are not likely to use digital health very soon.

*“My rheumatologist is like that; she tells me that she won’t invest in any digital infrastructure here and I know five, six, seven rheumatologists who are of the same age.”*

*(Patient representative I)*

According to the patient representative, monetary incentives or even sanctions for practitioners who decline to connect to digital infrastructure would be strategies to spread digitization in rheumatology care. According to the delegates, knowledge and digital health literacy are low among both practitioners and patients.

### 3.3. Adaptation to Different Environments of Rheumatology Care

Throughout the fishbowl discussion, participants agreed that digital health can complement current rheumatology care, but not replace face-to-face appointments. Digital health tools should be directed by medical practitioners. Diagnosis as well as important therapy decisions should be carried out by rheumatologists after a thorough clinical examination. Diagnosis, exclusively based on digital health, was strongly rejected by the discussants:

*“Initial diagnosis is only possible by the rheumatologist in direct physical contact and after detailed clinical examination. And then later on, you can talk about how the patient can be followed up by the internet or video consultation or anything else. That’s my opinion at least.”*

*(Rheumatologist III)*

*“Hey, a qualified initial diagnosis should please, please, please be made by a qualified rheumatologist. I think that is the consensus here in the panel.”*

*(Patient representative I)*

However, on the other side, participants mentioned different environments of rheumatology care, where digital healthcare could contribute. These are medical history, follow-up appointments, and disease monitoring:

*“Many patients have no idea what kind of medication they are taking, what kind of therapy they actually want, and I often spend most of my time gathering all this information. Electronic patient records could be a huge relief and I think that it is the key to speed up digitization.”*

*(Rheumatologist II)*

*“Yes, I am well-adjusted and my CRP is fine. But with new digital tools, for example the app that [name of a digital health application] is going to launch in the area of disease monitoring or something like [name of another application] … new solutions for disease monitoring are entering the market, which will contribute to maintaining the ability to work.”*

*(Patient representative I)*

Participants of the fishbowl discussion also highlighted the importance of digital approaches to ensure adequate and safe rheumatology care during the COVID-19 pandemic. They believe that secure and validated information on the internet can help people access adequate rheumatology care. Most importantly, this applies to the therapy of rare diseases.

### 3.4. Potentials of Digital Rheumatology Implementation

In regard to the healthcare system (macro level), the discussants highlighted the German Digital Health Act, which is anticipated to contribute to a digital transition of healthcare in the coming years:

*“It has been passed and eight months later the first products are about to be approved. I think that is the speed of light for the health system and shows again how the legislator wants to support this transition.”*

*(Digital product developer I)*

To increase the knowledge of medical practitioners and patients with regard to digital health services, the discussants call for education and information campaigns:

*“It can only be done with education and information campaigns. How should patients and practitioners receive the information, that there is the possibility of prescribing apps … and which ones are available … and how good they are?”*

*(Digital product developer II)*

Despite this momentum, the benefits of digital health applications still have to be proven:

*“Digitization is not positive in itself, but it might be positive in its effect and this effect still has to be proven, in other words, evaluated. And in the digitization law and specifically also for the introduction of apps, there is still a necessity to prove the benefit.”*

*(Rheumatologist I)*

According to the participants, a broader spectrum of rheumatology care services (meso level) will be available through digital transition. This provides the opportunity to effectively allocate the scarce human resources available in rheumatology care. The effective use of digital health in rheumatology can be supported by the innovative medical professional associations and active patient organizations:

*“How do the individual specialist areas position themselves in regard to digitalization and apps? I think that the rheumatologists—to praise them once again—are pretty much at the forefront here, as is the group of Young Rheumatologists and the Digital Commission. Ultimately, I consider it very state of the art to deal with the topic in this way.”*

*(Digital product developer II)*

In addition, digital health was also seen as a way to enhance the attractiveness of rheumatology as a profession in general:

*“I think this is precisely where digital aids could offer a very exciting aspect and expansion: To the extent that the job description of a rheumatologist becomes much more interesting and attractive when one knows that one can deal with digital, innovative tools in the treatment of patients.”*

*(Digital product developer I)*

On the micro level, respectively, individual level, patients and physicians could benefit from an improved accessibility to rheumatology care and the reduction of “unnecessary” appointments:

*“Because I also think it might be quite pleasant for patients to do without one or two unneeded doctor’s appointments.”*

*(Rheumatologist III)*

*“As long as things are fine, I don’t need to travel through the city to a non-accessible doctor’s office, anyways.”*

*(Patient representative I)*

Since much of the relevant information can be provided digitally in advance, the digital transition could increase consultation time:

*“Digitization allows us to have more time to talk. That is the quality of care, which is also increasing, because if I have already answered all the questions asked beforehand, then I have maybe five more minutes in the conversation with the patient, and that is an essential five minutes of conversation to improve the perceived care.”*

*(Rheumatologist I)*

### 3.5. Barriers to Digital Rheumatology Implementation

At the macro level, the discussants identified limited digital infrastructure and equipment as barriers to digital health transformation in Germany:

*“In many parts of society, that not only include special circumstances, but also students and the general population, from patients to doctors, who simply do not have the technical equipment. And I don’t even want to mention the 5G network, which is also not available in Germany.”*

*(Rheumatologist IV)*

On the meso level, the discussants pointed out that the current remuneration systems in rheumatology care are not designed for digital approaches and does not incentivize them:

*“… also the remuneration of digital services. And I think this is the challenge right now, especially in rheumatology … set it up digitally and get away from ‘well, I only want to see the patients who also bring me the money, but I try to care for the patients according to their needs with digital support’.”*

*(Digital product developer I)*

This results in a barrier on the micro level at the same time. Practitioners could suffer financial losses through the use of digital health. Furthermore, digital health could lead to a wealth gap on the part of patients, which results in health opportunities being distributed unequally, a digital divide in healthcare:

*“We also have to consider that digitalization creates a wealth gap: people who cannot afford large contracts, good mobile phones, good tablets, do not have good access. And this is also evident in telemedicine and applications, where these systems are not being used. Thus, I believe that we also have to consider the social aspect.”*

*(Rheumatologist I)*

Finally, the confusing numbers of available digital tools, limited individual knowledge and interest concerning digital health represent a barrier to digital transition in daily rheumatology practice:

*“These apps that we are supposed to recommend—to pick up on this—I personally have a very, very hard time with them in my day-to-day practice. Surprisingly, not a single patient has asked me for an app so far. So, all this information is not yet there and then I once again ask you: Why has no patient asked for it yet?”*

*(Rheumatologist V)*

## 4. Discussion

We performed a qualitative study using a virtual fishbowl discussion on digital transition in rheumatology with 476 participants at the first virtual conference of German Society for Rheumatology 2020. The qualitative approach provides a unique insight on the status quo of digital rheumatology from the perspectives of patient representatives, rheumatologists, and digital product developers.

The participants revealed a positive view toward digital transition. They identified digital health as a valuable addition to current rheumatology care, mentioning various use cases that could support existing services: electronic patient records, digital health applications (diagnosis assistance/monitoring), and implementation of a health information portal. The participants of the fishbowl discussion rejected digital approaches if these were decoupled from the traditional healthcare system. Final diagnosis and therapeutic decisions should exclusively be made by rheumatologists. Digital health should be integrated into rheumatology care routines but must not entirely eradicate personal patient–doctor interaction [2]. Digital rheumatology is considered as supportive regarding anamnesis, follow-up appointments, monitoring, and (validated) information on services and diseases. The discussants highlighted patients with stable disease courses as potential telemedicine users. This is in line with the research by Kulcsar et al. [19]. The importance of digitally provided information for patients with rare diseases was stressed. The accelerating and connecting effect of digitization—“digital crowdsourcing”—to inform patients is consistent with previous results by Krusche et al. [20] and Ruffer et al. [21]. On the other hand, with regard to the medical practitioners, less technophile doctors would not be likely to adopt digital health in their clinical routines, according to the discussants. Similarly, low eHealth literacy and skepticism was observed among older rheumatic patients [22]. Overall, knowledge on digital health is still very limited among practitioners and patients according to the discussants. Previous survey data [23,24] confirm this lack of knowledge as a main barrier. It also appears that in particular highly affected patient groups are significantly less interested in eHealth [25]. On the macro level, the participants in the fishbowl discussion pointed out that German Digital Health Care Act as well as education and information campaigns could foster digital transition. The weak digital infrastructure and equipment in Germany is a barrier from the discussants’ perspectives. Regarding rheumatology care as a whole (meso level), digital transition could support effective allocation of scarce rheumatology workforce and enhance the attractiveness of rheumatology as a profession. It has been demonstrated that telemedicine-based monitoring is not clinically inferior to standard follow-up [26]. Furthermore, it has been demonstrated that digitally complemented lectures increase students’ interest in rheumatology [27]. The participants identified inadequate remuneration systems and a lack of incentives as a potential barrier to digital health on the meso level. This underlines the high relevance of innovative and sustainable funding systems for large-scale implementation of eHealth [28]. On the individual patient–practitioner level (micro level), digital health could improve accessibility of rheumatology care services, increase individual consultation time, and at the same time enable the care of more patients overall by time savings in clinical practice. This is in line with recent qualitative findings that point to the potential of digital health contributing to patient-centered care in rheumatology [29]. The opportunities of digital health in rheumatology care are contrasted by possible financial losses among practitioners and limited knowledge about digital health as barriers. Finally, the influence of the digital transition on social disparities and the allocation of health opportunities is controversially discussed and needs to be intensively observed [30,31,32].

Study findings could have direct implications for informing guidelines on remote care in rheumatology and can help to successfully implement digital health tools in the management of rheumatic diseases. A main strength of this study is the inclusion of potential users of digital health and in particular patient representatives. To the best of our knowledge, this is the first qualitative study in rheumatology that included the three stakeholders: patients, rheumatologists, and digital product developers. We also highly recommend the virtual fishbowl format [10], as it is an outstanding option for multi-perspective, interactive discussions and, during COVID-19, a solid alternative to physical face-to-face debates. Compared to other discussion formats, i.e., panel discussions or large debates, fishbowl discussions encourage active participation of non-experts and enable the inclusion of multiple perspectives [12].

The study design has several limitations. The study reports on the content of one 54-min discussion session. Because conference delegates chose to participate in the discussion based on their interest in the topic, a self-selection bias and a rather positive attitude toward digital health is likely to exist. The two introductory key notes on digital services in rheumatology might have positively biased the subsequent fishbowl and our results. Although fishbowl is an inclusive, participatory discussion technique, not all attendees participated equally in the discussion. The discussion was held at a virtual medical conference including mainly rheumatologists. The patient perspective is underrepresented, as mostly physicians expressed their views and only one patient representative actively took part in the discussion.

## 5. Conclusions

German patients, rheumatologists, and digital health developers expressed a generally positive attitude toward the digital health transition in rheumatology. It could contribute to effective allocation of scarce resources, improve accessibility, and enhance the attractiveness of rheumatology as a profession. Digital health may certainly complement rheumatology care, but (final) diagnosis and key decisions are to remain in the hands of rheumatologists, according to the participants of the fishbowl discussion. Patients and rheumatologists need more information and education to successfully implement digital health tools into clinical routine.

## Data Availability

All data relevant to the study are included in the article. For further questions regarding the reuse of data, please contact the corresponding author (felix.muehlensiepen@mhb-fontane.de).

## Supplementary Materials

The following are available online at https://www.mdpi.com/1660-4601/18/5/2636/s1: Table S1: COREQ Checklist.
